# Systematic profiling of invasion‐related gene signature predicts prognostic features of lung adenocarcinoma

**DOI:** 10.1111/jcmm.16619

**Published:** 2021-05-31

**Authors:** Ping Yu, Linlin Tong, Yujia Song, Hui Qu, Ying Chen

**Affiliations:** ^1^ Department of Medical Oncology The First Hospital of China Medical University Shenyang China; ^2^ Key Laboratory of Anticancer Drugs and Biotherapy of Liaoning Province The First Hospital of China Medical University Shenyang China; ^3^ Liaoning Province Clinical Research Center for Cancer Shenyang China

**Keywords:** invasion genes, LUAD, molecular subtype, multi‐gene signature, TCGA

## Abstract

Due to the high heterogeneity of lung adenocarcinoma (LUAD), molecular subtype based on gene expression profiles is of great significance for diagnosis and prognosis prediction in patients with LUAD. Invasion‐related genes were obtained from the CancerSEA database, and LUAD expression profiles were downloaded from The Cancer Genome Atlas. The ConsensusClusterPlus was used to obtain molecular subtypes based on invasion‐related genes. The limma software package was used to identify differentially expressed genes (DEGs). A multi‐gene risk model was constructed by Lasso‐Cox analysis. A nomogram was also constructed based on risk scores and meaningful clinical features. 3 subtypes (C1, C2 and C3) based on the expression of 97 invasion‐related genes were obtained. C3 had the worst prognosis. A total of 669 DEGs were identified among the subtypes. Pathway enrichment analysis results showed that the DEGs were mainly enriched in the cell cycle, DNA replication, the p53 signalling pathway and other tumour‐related pathways. A 5‐gene signature (KRT6A, MELTF, IRX5, MS4A1 and CRTAC1) was identified by using Lasso‐Cox analysis. The training, validation and external independent cohorts proved that the model was robust and had better prediction ability than other lung cancer models. The gene expression results showed that the expression levels of MS4A1 and KRT6A in tumour tissues were higher than in normal tissues, while CRTAC1 expression in tumour tissues was lower than in normal tissues. The 5‐gene signature prognostic stratification system based on invasion‐related genes could be used to assess prognostic risk in patients with LUAD.

## 1 INTRODUCTION

Lung cancer is the most common cause of cancer‐related death worldwide.^1^ About 85% of lung cancers are non–small‐cell lung carcinomas,^2^ which can be divided into 3 histological subtypes, with lung adenocarcinoma (LUAD) being the most common.^3^ Major risk factors for LUAD include smoking, genetic factors, diet, alcohol consumption, and exposure to ionizing radiation and environmental pollutants.^4^, ^5^ As the early symptoms of LUAD are not obvious, most patients with LUAD are diagnosed with advanced stages, though metastasis occurs earlier, and the 5‐year overall survival (OS) rate is less than 20%.^6^ Although progress has been made in terms of early diagnostic methods, chemotherapy, radiotherapy, and surgical diagnosis and treatment options in recent years, the prognoses of patients with LUAD remain poor.^7^

At present, lung cancer treatment mainly depends on histological type and clinical stage, but due to the high heterogeneity of LUAD, even patients with the same histological type and clinical stage of LUAD have different prognoses, so the classification of LUAD based on high‐throughput sequencing data is of great significance for individualized and accurate LUAD treatment.

In recent years, the use of high‐throughput sequencing technology to detect a large number of gene expression changes, combined with the use of bioinformatics methods to systematically analyse tumour‐related genes and their regulatory mechanisms, has become an important research means in functional genomics, and it has been widely used to screen potential tumour biomarkers.^8^, ^9^, ^10^ In their research of LUAD, Krzystanek et al^11^ identified a 7‐gene signature (*ADAM10*, *DLGAP5*, *RAD51AP1*, *FGFR10P*, *NCGAP*, *KIF15* and *ASPM*) by analysing microarray data of early LUAD from the Gene Expression Omnibus (GEO) database, and they found significant differences in survival and prognosis among these genes. Li et al^12^ constructed a 5‐gene signature, which was closely related to the tumour microenvironment, by using the GSE103584 data set. The 13‐gene signature constructed by He et al^13^ with metabolism‐related genes was helpful to predict the prognoses of patients with LUAD. Han et al^14^ constructed a multi‐gene signature based on tumour‐infiltrating B lymphocyte–specific genes to predict the clinical outcome of radiotherapy and immunotherapy in patients with LUAD. Li et al^15^ used a 6‐gene signature to predict the prognoses of patients with LUAD.

In this study, we identified molecular subtypes of LUAD based on tumour invasion–related genes by using gene expression data from public databases, such as The Cancer Genome Atlas (TCGA) and GEO, for the first time. We evaluated the relationships between the molecular subtypes and prognosis and clinical features. The prognostic risk model based on differentially expressed genes (DEGs) between the LUAD subtypes could be used to evaluate LUAD prognosis. In addition, the nomogram we constructed could be used to help clinical decision‐making and prognosis judgement.

## 2 METHODS AND MATERIALS

### 2.1 Data download and preprocessing

RNA‐sequencing data and clinical follow‐up information for LUAD were downloaded from the TCGA database. The GSE31210 chip data set containing survival time information was downloaded from the GEO database.

Invasion‐related genes were obtained from the *CancerSEA* website,^16^ which contains 97 genes (Table S1).

The TCGA‐LUAD RNA‐sequencing data were preprocessed as follows: (1) the samples with no clinical follow‐up information were removed; (2) the samples with no survival time information were removed; (3) the samples with no status information were removed; (4) the Ensemble IDs were transformed into gene symbols; and (5) the median expression of multiple gene symbols was obtained.

The GEO data were preprocessed as follows: (1) the samples with no clinical follow‐up information were removed; (2) the samples with no survival time or status information were removed; (3) the probes were converted into gene symbols; (4) the probes were mapped to multiple genes, and the probes were deleted; and (5) the median expression of multiple gene symbols was obtained.

After preprocessing, there were 500 TCGA‐LUAD samples and 126 GSE31210 data set samples. The clinical statistics of the samples can be found in Table 1.

**TABLE 1:** Sample information

Clinical features	TCGA‐LUAD	GSE31210
OS		
0	318	191
1	182	35
Gender		
Female	270	121
Male	230	105
Age		
≤60	157	0
>60	343	226
T Stage		
T1	167	
T2	267	
T3	45	
T4	18	
N Stage		
N0	324	
N1	94	
N2	69	
N3	2	
M Stage		
M0	332	
M1	24	
Stage		
I	268	168
II	119	58
III	80	0
IV	25	0
Smoking history		
1	71	
2	119	
3	129	
4	163	
5	4	

### 2.2 Consistent clustering

The expression levels of 97 invasion‐related genes were extracted from the TCGA expression profiles, and genes related to LUAD prognosis were obtained by univariate Cox analysis using the coxph function in R (*P* < .01). ConsensusClusterPlus (V1.48.0) was used to cluster the samples consistently according to significant genes from the single‐factor Cox analysis (parameters: reps = 100, pItem = 0.8, pFeature = 1, distance = Minkowski). Pam and Minkowski distances were used as the clustering algorithm and distance measure, respectively.

### 2.3 Identification of differentially expressed genes

The DEGs of different molecular subtypes were calculated by using the limma package in R.^17^ The DEGs were filtered according to the threshold of FDR <0.01 and |log2fc| > 1, and then, volcano maps were plotted.

### 2.4 GO and KEGG enrichment analyses

The results of Gene Ontology (GO) and Kyoto Encyclopedia of Genes and Genomes (KEGG) functional enrichment analyses using the DAVID database showed that *P* < .05 was statistically significant. The results were visualized by using the ggplot2 package in R (V3.5.3).

### 2.5 Calculation of immune scores

Stromal, immune and estimate scores were calculated by using the ESTIMATE package in R. Ten immune cells were evaluated by using MCPcounter, and 28 immune cells were evaluated by using single‐sample gene set enrichment analysis (ssGSEA) with the GSVA package in R.^18^

### 2.6 Construction of prognostic risk model

The 500 samples in the TCGA data set were divided into training and validation cohorts. To avoid the influence of random assignment bias on the stability of subsequent modelling, all samples were put back into the random grouping 100 times in advance, and the group sampling was carried out according to the training cohort‐to‐validation cohort ratio of 1:1. The final training cohort contained 250 samples, and the final validation cohort contained 250 samples.

### 2.7 Univariate and multivariate Cox regression analyses

The survival *coxph* function in R was used to analyse the DEGs among the molecular subtypes and the survival data through univariate Cox proportional hazard regression. We selected *P* < .001 as the filter threshold, and genes related to prognosis were obtained. The R package *glmnet* was used to perform Lasso regression on the DEGs and prognosis‐related genes to compress the risk model to reduce the number of genes.^19^ The step method in the stats package in R starts from the most complex model and reduces the number of parameters by deleting 1 variable in turn. The smaller the step value, the more superior the model. This means that the fitting degree of the model is better with fewer parameters. The number of genes in the risk model was further reduced by using the Akaike information criterion (AIC) algorithm.

### 2.8 Gene set enrichment analysis

To observe the relationships between risk scores and biological functions in different samples, the gene expression profiles of these samples were selected for ssGSEA using the GSVA R software package. The ssGSEA scores of each function corresponding to each sample were obtained by calculating the scores of different functions for each sample. The correlations between these functions and risk scores were further evaluated. Functions with correlations >0.4 were selected.

### 2.9 Construction and verification of nomogram

Nomograms can show the results of risk models intuitively and effectively, and they are convenient to use to predict outcome. A nomogram uses line length to indicate the degree of influence of different variables and different values of variables on outcome. Based on the results of single‐ and multi‐factor analyses, a nomogram model was constructed.^20^

### 2.10 Gene expression in pan‐carcinoma

We downloaded 6 immune‐infiltrating cell scores from 33 cancers in the TIMER database (https://cistrome.shinyapps.io/timer/), and we analysed the expression of 5 genes in these 33 cancer tissues.

### 2.11 Clinical expression of genes in the Oncomine and GEO cohorts

Oncomine (http://www.oncomine.org) is a gene chip‐based database and integrated data‐mining platform. In this study, we set the screening criteria as follows: (a) cancer type: LUAD; (b) analysis type: cancer vs normal analysis; and (c) threshold criteria: *P* < .05, fold change >1.5 and gene rank = top 10%. The LUAD cohort was downloaded from the GEO database, and the ggplot2 R package was used to visualize the expression levels of 5 genes in the LUAD data set.

### 2.12 Immunohistochemistry and protein‐level validation

The Human Protein Atlas (HPA) provides information on the tissue and cell distributions of 26 000 human proteins. We explored protein levels relating to the 5 genes in normal lung and tumour tissues.

## 3 RESULTS

### 3.1 Study flow chart

**FIGURE 1:**
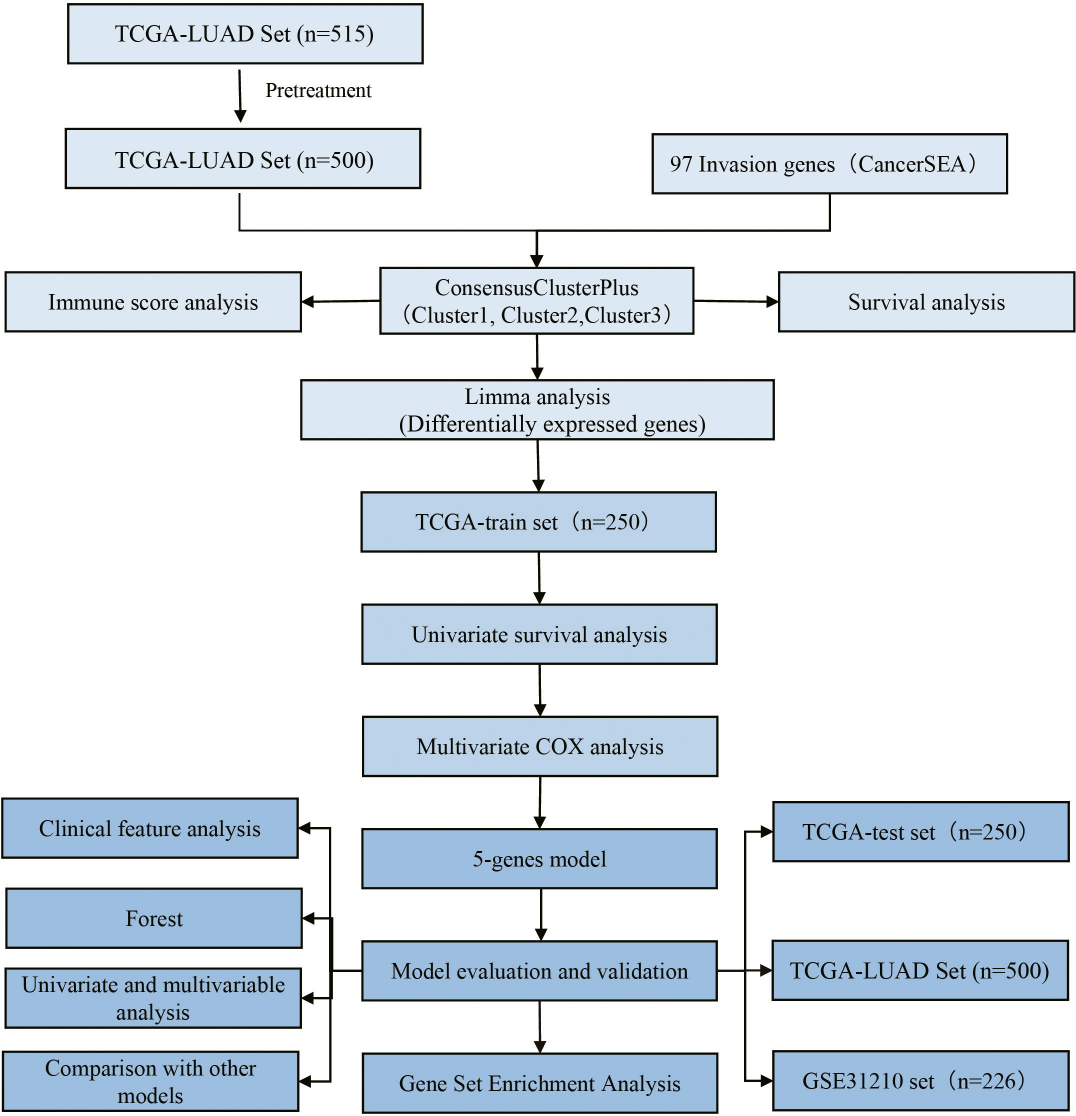
Flow chart of prognosis‐ and invasion‐related features in lung adenocarcinoma samples

To make the research easier for readers to understand, we drew a methodology flow chart (Figure 1).

### 3.2 Molecular typing of LUAD based on invasion‐related genes

**FIGURE 2:**
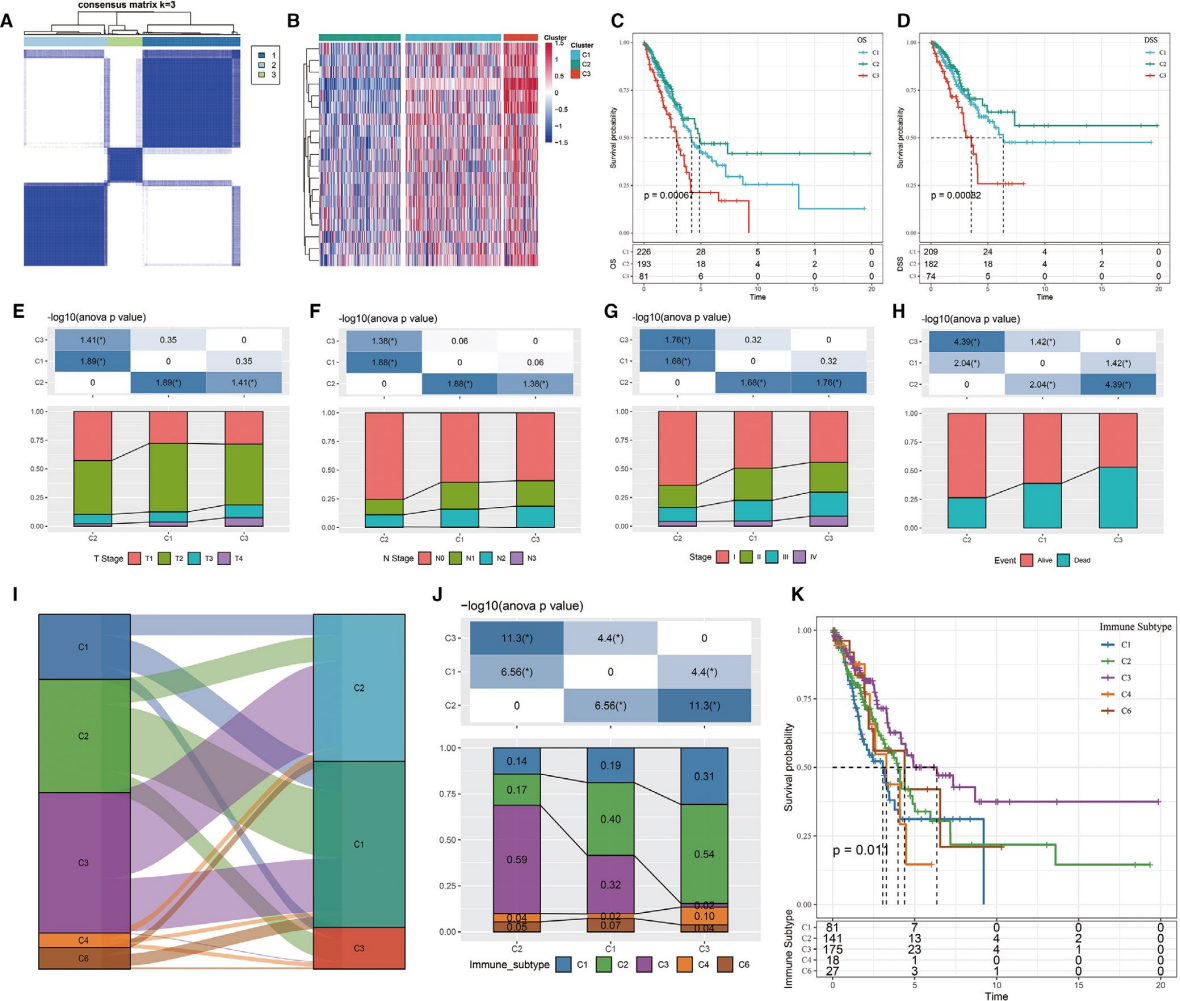
A, Cluster thermograms of samples with consistent clusters of k = 3. B, Cluster thermograms of prognosis‐related invasion genes. C, Survival curves of TCGA lung adenocarcinoma samples with different molecular subtypes. D, TCGA lung adenocarcinoma samples according to different molecular subtypes. E‐H, Distribution comparison of clinical features among the 3 subtypes of the TCGA data set. I, Sanki map of molecular subtypes compared with existing molecular immune subtypes. J, Distribution of molecular subtypes compared with existing immune subtypes. K, Survival curves of the molecular immune subtypes

Through univariate Cox analysis of the 97 invasion‐related genes in the TCGA expression profile data, 19 genes were found to be associated with LUAD prognosis (*P* < .01; Table S2). Consistent cluster analysis showed that the samples could be clustered at *k* = 3 (Figure 2A). The expression levels of the invasion‐related genes in the 3 subtypes are shown in Figure 2B. These levels were different among the C1, C2 and C3 subtypes. Most of the genes were highly expressed in the C3 subtype and lowly expressed in the C2 subtype. We further analysed the relationships between the 3 subtypes and prognosis. The results showed that there were significant differences between the 3 subtypes. The prognoses of patients with the C2 subtype were the best, and those of patients with the C3 subtype were the worst (log‐rank *P* < .05; Figure 2C,D).

### 3.3 Identification and functional analysis of DEGs among subtypes

The DEGs between C1, C2 and C3 were identified by using the *limma* package in R. The volcano map of the DEGs between C1 and C3 is shown in Figure S1A; there were 98 up‐regulated genes and 123 down‐regulated genes. The volcano map of the DEGs between C1 and C2 is shown in Figure S1B; there was 1 up‐regulated gene and 4 down‐regulated genes. The volcano map of the DEGs between C2 and C3 is shown in Figure S1C; there were 389 up‐regulated genes and 267 down‐regulated genes.

A total of 669 DEGs between C1/C2, C2/C3 and C1/C3 were obtained, and these DEGs were further analysed by KEGG pathway and GO functional enrichment analyses using the *WebGestaltR* (V0.4.2) software package in R. The biological functions of the top 10 genes enriched in biological processes (Figure S1D), cellular components (Figure S1E) and molecular functions (Figure S1F) were visualized. The KEGG pathway analysis results showed that the DEGs were significantly enriched in the cell cycle, DNA replication, p53 signalling pathway, microRNAs in cancer, small‐cell lung cancer and other tumour‐related pathways (Figure S1G).

### 3.4 Clinical correlations of molecular subtypes and comparison with existing subtypes

The distributions of different clinical features among the C1, C2 and C3 subtypes were compared. The results showed that there were significantly more C2 patients than C1 and C3 patients in the T1, N0 and Stage I samples, while there were significantly fewer C2 patients than C1 and C3 patients in the T2, N1 and Stage II samples (Figure 2E‐G). The number of survivors in the C2 group was significantly higher than in the C1 and C3 groups (Figure 2H). These results confirmed that patients with the C2 subtype had the best prognoses.

Previous studies have analysed 33 cancers in the TCGA database. These studies clustered non‐blood tumours into 6 immune subtypes based on the distributions of various features, such as macrophages, immune‐infiltrating lymphocytes, transforming growth factor‐beta response, interferon‐γ response and wound healing; these subtypes include C1 (wound healing), C2 (INF‐**γ** predominance), C3 (inflammation), C4 (lymphocyte depletion), C5 (immunological silencing) and C6 (transforming growth factor‐beta predominance), among which C1 and C6 have been associated with poor prognosis.^21^ By comparing the molecular subtypes with these immune subtypes, it was found that most LUAD patients in the TCGA data set belonged to the C1, C2 and C3 immune subtypes (about 89.5%), and there were no patients with the C5 immune subtype in the LUAD TCGA data set (Figure 2I). By comparing the distributions of the molecular and immune subtypes, it was found that patients with the C3 molecular subtype showed the highest proportion of the C2 immune subtype, reaching 54% (Figure 2J). The proportion of the C2 immune subtype among the C2 molecular subtype was lower, and the proportion of the C3 immune subtype was higher than that of the C3 molecular subtype. The survival curve analysis results showed that there were significant differences in OS among the immune subtypes (*P* < .05; Figure 2K). These results suggested that the prognosis of the C3 immune subtype was better than that of the C2 immune subtype.

### 3.5 Comparison of immune scores among subtypes

**FIGURE 3:**
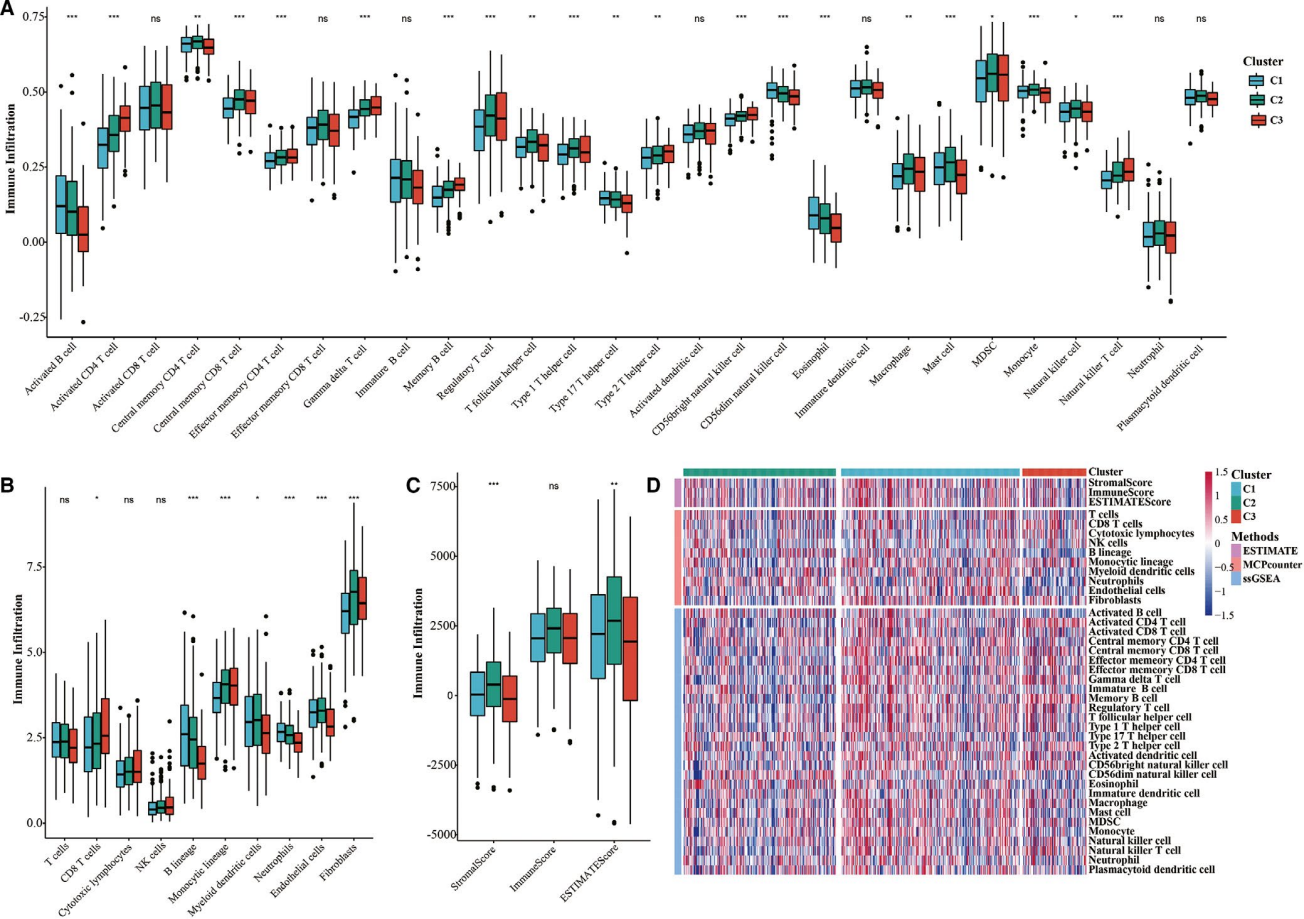
A, Comparison of the ssGSEA immune scores among the subtypes of the TCGA data set. B, Comparison of the MCPcounter immune scores among the subtypes of the TCGA data set. C, Comparison of the estimate immune scores among the subtypes of the TCGA data set. D, Comparison of all 3 immune score types among the molecular subtypes of the TCGA data set

The relationships between the molecular subtypes of the TCGA data set and immune scores were identified by using the ESTIMATE software package in R, MCPcounter, and the ssGSEA method in the GSVA package. The results showed that there were significant differences in immune scores among the different subtypes (Figure 3A‐C). The heat map of immune scores among the 3 subtypes is shown in Figure 3D.

### 3.6 Construction of risk model

The 500 samples in the TCGA data set were grouped according to the training set‐to‐validation set ratio of 1:1, and the univariate Cox proportional hazard regression model method was used to evaluate the 669 DEGs between the molecular subtypes. A total of 29 genes were found to be associated with prognosis (Table S3). Lasso regression was used to further compress the 29 genes. The trajectory of each independent variable is shown in Figure S2A. As lambda decreased, the number of independent variable coefficients tending to 0 increased. We used 10‐fold cross‐validation to build the model and analysed the confidence interval (CI) under each lambda (Figure S2B). When lambda equalled 0.003518527, the model was considered optimal, and 12 genes (*KRT6A*, *MELTF*, *IL20RB*, *PLEK2*, *LOXL2*, *IRX5*, *SLC16A11*, *FAM189A2*, *ITGA6*, *PKP2*, *MS4A1* and *CRTAC1*) were selected as target genes. The AIC algorithm was used to further compress these 12 target genes, and 5 target genes (*KRT6A*, *MELTF*, *IRX5*, *MS4A1* and *CRTAC1*) were finally obtained.

The Kaplan‐Meier curves of the 5 genes are shown in Figure S2C‐G. The 5 genes could be divided into 2 groups with high and low risk (*P* < .05). The final 5‐gene signature formula was as follows: RiskScore = 0.08073881 * KRT6A + 0.18237095 * MELTF − 0.17903164 * IRX5 − 0.26862737 * MS4A1 − 0.09946249 * CRTAC1.

**FIGURE 4:**
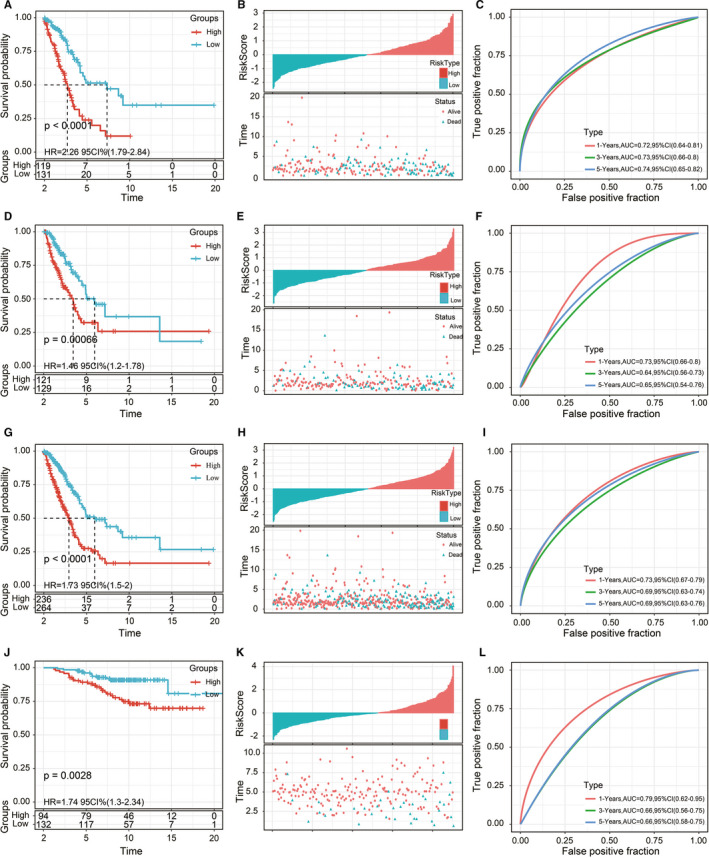
A, Survival curves between the 2 risk groups based on the 5‐gene signature classification. B, Distributions of risk scores and survival status based on the 5‐gene signature in the TCGA training cohort. C, ROC curve of the 5‐gene signature classification in the TCGA training cohort. D‐F, Survival curves between the 2 risk groups, distributions of risk scores and survival status, and the ROC curve of the 5‐gene signature in the TCGA testing cohort. G‐I, Survival curves between the 2 risk groups, distributions of risk scores and survival status, and the ROC curve of the 5‐gene signature in the entire TCGA cohort. J‐L, Survival curves between the 2 risk groups, distributions of risk scores and survival status, and the ROC curve of the 5‐gene signature in the GSE31210 cohort

Risk scores were further converted into Z‐scores. Samples with scores >0 were divided into the high‐risk group, and samples with scores < 0 were divided into the low‐risk group. A total of 119 samples were divided into the high‐risk group, and 131 samples were divided into the low‐risk group. The survival curve results showed that there was a significant difference in prognosis between the 2 groups (*P* < .0001; Figure 4A).

The risk score distributions of the samples were calculated according to expression levels and then plotted (Figure 4B). The survival times of the samples with high‐risk scores were significantly shorter than those of the samples with low‐risk scores, suggesting that samples with high‐risk scores had worse prognoses. The timeROC software package in R was used to analyse the prognostic classification efficiency of risk scores. The model had a large area under the curve (AUC) at 1, 3 and 5 years; the 1‐year AUC was 0.72, and the 5‐year AUC was 0.74 (Figure 4C).

### 3.7 Verification of risk model robustness in internal and external data sets

The robustness of the model was verified by the internal data set (TCGA validation set and all data sets) and external data set (GSE31210 data set). In all data sets, the same model and coefficients as those in the training set were used. The survival curve showed significant differences between the high‐ and low‐risk groups in the verification set and all data sets (Figure 4D,G). The risk score of each sample was calculated according to gene expression, risk score distributions were plotted in TCGA internal validation set and all data sets in Figure 4E,H. The classification efficiencies of prognosis prediction at 1, 3 and 5 years in the TCGA testing cohort and entire TCGA cohort are shown in Figure 4F,I, respectively. The 1‐year AUC reached 0.73 in both data sets.

Z‐score transformation of risk scores was performed in GSE31210 data set. Samples with risk scores >0 after Z‐score transformation were divided into the high‐risk group, and samples with risk scores <0 after Z‐score transformation were divided into the low‐risk group. This resulted in 94 samples in the high‐risk group and 132 samples in the low‐risk group. The survival curve showed a significant difference between the high‐ and low‐risk groups (*P* = .0028; Figure 4J).

The risk score distribution of the samples in the GSE31210 cohort was consistent with that of the training set (Figure 4K). Receiver operating characteristic (ROC) analysis showed that the 1‐year AUC reached 0.79 (Figure 4L).

### 3.8 Relationships among risk scores, clinical features and molecular subtypes

**FIGURE 5:**
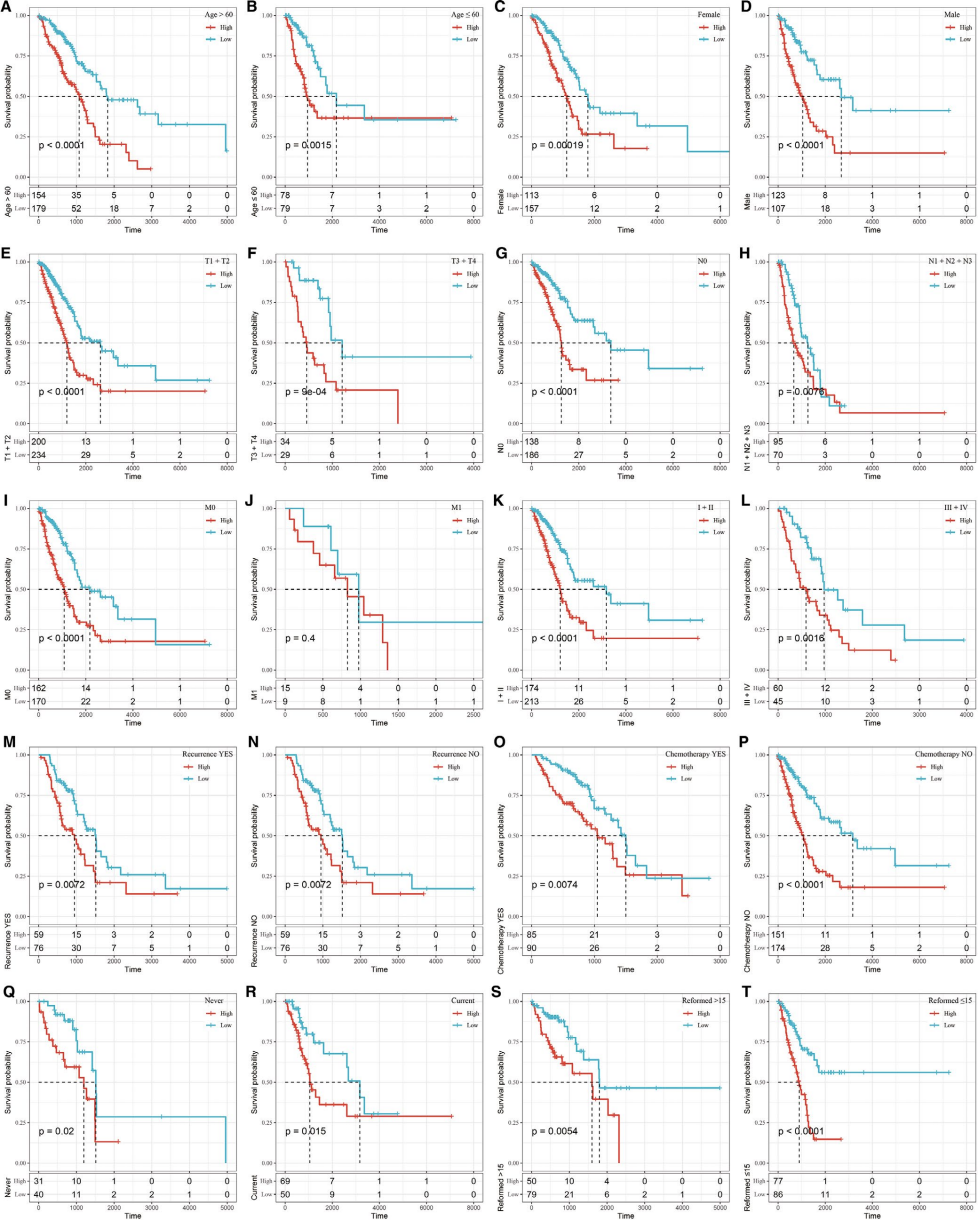
Prognostic performance of the 5‐gene signature in terms of different clinical features

Survival analysis of different clinical subgroups was carried out based on risk scores. The results showed that the 5‐gene signature could significantly distinguish between age, sex, tumour/node/metastasis (TNM) stage, stage, recurrence, chemotherapy and smoking status (current smoker, never smoked, reformed smoker) samples into high‐ and low‐risk groups (*P* < .05; Figure 5A‐T). The 5‐gene signature could not divide the M1 samples into 2 groups with significant prognostic difference, which may be due to the small number of M1 samples (*P* > .05; Figure 5J). In general, our model could be used as a prognostic marker for different clinical subgroups if the sample size was appropriate.

Risk score distributions in terms of different clinical features were also assessed. The results showed that there were no significant differences in terms of age or stage (*P* > .05; Figure S3B,E). Risk scores showed significant differences in terms of sex (female, male), T stage (T1, T2, T3, T4), N stage (N0, N1, N2, N3), stage (Stage I, Stage II, Stage III, Stage IV) and smoking status (current smoker, never smoke, reformed smoker) (*P* < .05; Figure S3A,C,D,F,G). We also compared risk scores among the 3 subtypes (C1, C2 and C3). The results showed that the risk scores of C3 subtype samples with poor prognosis were significantly higher than those of C2 subtype samples with good prognosis (Figure S3H), which further suggested that high‐risk scores were associated with poor survival outcome.

### 3.9 Relationships between risk scores and pathways

To observe the relationships between the risk scores and biological functions of different samples, GSEA was used to calculate the scores of different functions for each sample and correlations between these functions and risk scores. Correlation scores >0.4 were considered to show positive correlations. Nine pathways were positively correlated with risk scores, and 10 pathways were negatively correlated with risk scores (Figure S4A). The 19 most relevant KEGG pathways were selected for cluster analysis (Figure S4B) based on their enrichment scores. Tumour‐related pathways, such as KEGG_P53_SIGNALING_PATHWAY, KEGG_CELL_CYCLE, KEGG_MISMATCH_REPAIR and KEGG_DNA_REPLICATION, were activated as risk scores increased, while others, such as KEGG_ARACHIDONIC_ACID_METABOLISM, KEGG_GLYCEROPHOSPHOLIPID_METABOLISM and KEGG_ETHER_LIPID_METABOLISM, were deactivated as risk scores increased.

### 3.10 Construction of nomogram

**FIGURE 6:**
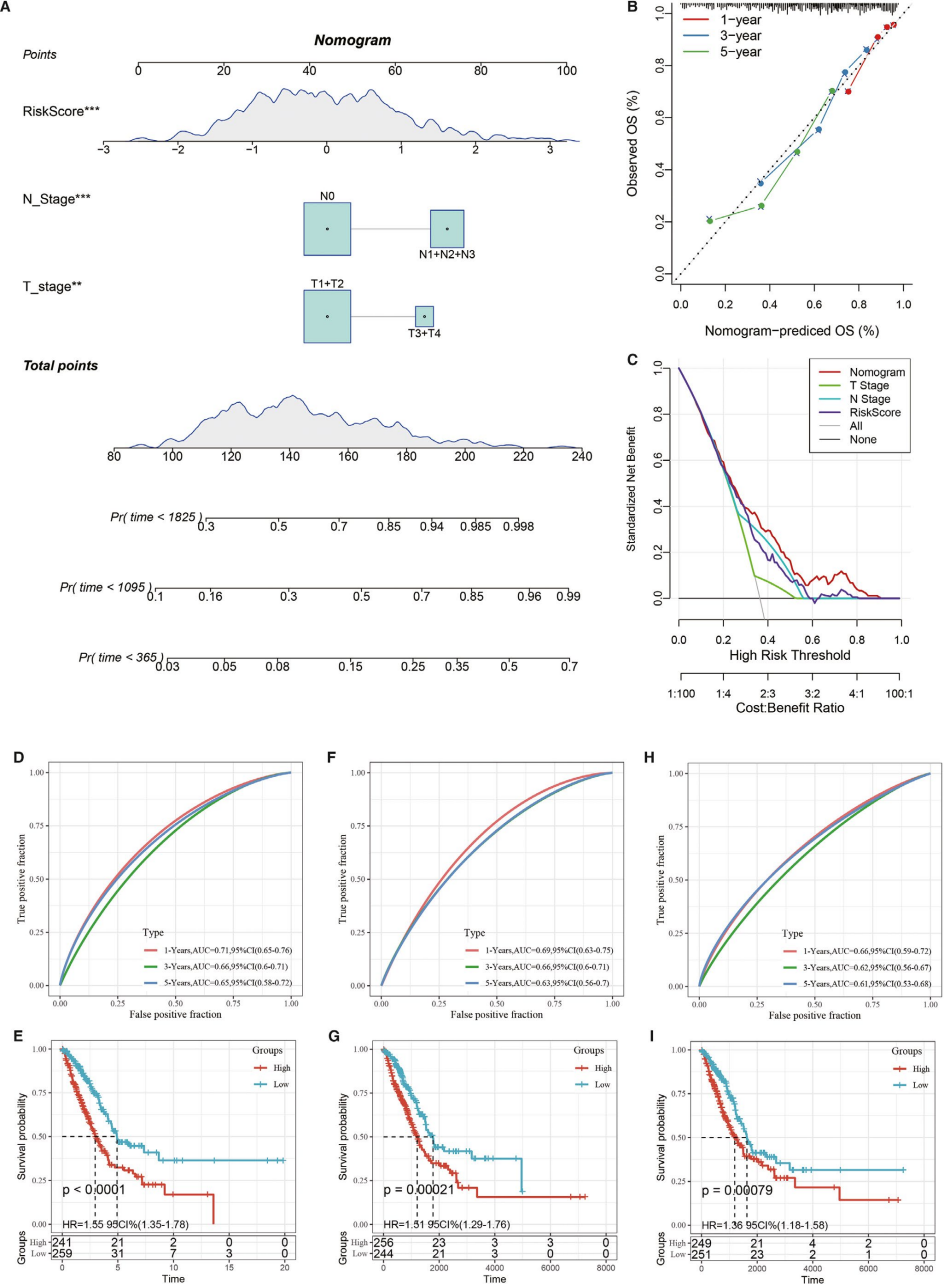
A, Construction of the nomogram model. B, Calibration curves at 1, 3 and 5 years using the nomogram. C, Decision curve analysis of age, M stage, clinical stage, risk score and nomogram results. D‐E, ROC curve of the 8‐gene signature risk model (Li) and the Kaplan‐Meier curves of the high‐ and low‐risk LUAD samples. F‐G, ROC curve of the 3‐gene signature risk model (Yue) and the Kaplan‐Meier curves of the high‐ and low‐risk LUAD samples. H‐I, ROC curve of the 3‐gene signature risk model (Liu) and Kaplan‐Meier curves of the high‐ and low‐risk LUAD samples

Univariate and multivariate Cox regression analyses were used to analyse the independence of the 5‐gene signature model in terms of clinical applications. Univariate analysis results showed that TNM stage, stage and risk scores were significantly correlated with survival time; multivariate Cox regression analysis results showed that risk scores (HR = 1.63, 95% CI = 1.34‐2.96, *P* < 1e−5) and N stage (HR = 1.99, 95% CI = 1.34‐2.96, *P* < .001) were independent prognostic risk factors (Figure S5A,B). A nomogram was constructed based on the significant variables of multiple factors (Figure 6A), and the results showed that risk scores had the greatest effect on survival prediction, suggesting that the 5‐gene signature was a good predictor of survival. Furthermore, by using calibration curves to evaluate the accuracy of the model (Figure 6B), it was observed that the calibration curves at 1, 3 and 5 years were close to the standard curve, suggesting that the model had good prediction performance. Moreover, decision curve analysis was used to evaluate the model's reliability (Figure 6C), and the results showed that the benefits of risk scores and the nomogram were significantly higher than those of the extreme curve, and the effect of the nomogram was higher than the effects of T stage, N stage and risk scores, which were close to the extreme curve, suggesting that risk scores and the nomogram had good clinical applicability.

### 3.11 Comparison of risk model with other models

To prove the superiority of our model, 3 risk models, including an 8‐gene signature (Li),^22^ a 3‐gene signature (Yue)^23^ and a 3‐gene signature (Liu),^24^ were chosen to compare with our 5‐gene signature. To make the models comparable, we calculated the risk score of each LUAD sample in the TCGA data set by the same method, and we evaluated the ROC curve of each model. Z‐score transformation of risk scores was performed. The samples with risk scores >0 after Z‐score transformation were divided into the high‐risk group, and samples with risk scores <0 after Z‐score transformation were divided into the low‐risk group. The survival curves were plotted. The results showed that all 3 models could significantly classify the high‐ and low‐risk groups into prognostic categories (Figure 6E,G,I). However, the AUCs of the ROC curves of the 3 models were lower than those of the 5‐gene signature at 1, 3 and 5 years in the TCGA data set (Figure 6D,F,H). These results showed that our model had good clinical predictive power.

### 3.12 Expression and prognosis of 5 genes in 33 pan‐cancers

The box diagram showed that *MS4A1* was significantly highly expressed in LUAD, HNSC and kidney renal clear cell carcinoma, while in bladder carcinoma, colon adenocarcinoma, KICH, and READ tumours, *MS4A1* was significantly lowly expressed (Figure S6A). Compared with normal samples, *KRT6A* and *MELTF* showed significantly high expression in most cancer types, including LUAD (Figure S6C), while *CRTAC1* was expressed lowly in most cancer types, including LUAD (Figure S6D, *IRX5* was significantly highly expressed in breast cancer, CHOL, colon adenocarcinoma, kidney renal papillary cell carcinoma, liver hepatocellular carcinoma and READ tumours, while in KICH, kidney renal clear cell carcinoma, lung squamous cell carcinoma and PRAD, *IRX5* was significantly lowly expressed (Figure S6E).

Furthermore, we used forest plots to show the prognostic significance of 5 genes in 33 cancer types of tumour tissues. The results showed that MS4A1 gene is a protective gene in most cancer types, and the high expression of MS4A1 gene has a better prognosis. In addition, CRTAC1 and IRX5 are also protective genes in LUAD; MELTF gene is a risk gene in most cancer types, the prognosis of patients with high expression of MELTF gene is worse; at the same time, patients with high expression of KRT6A are also associated with poor prognosis (Figure S7A‐E).

### 3.13 Clinical validation of 6 genes in terms of protein and mRNA expression

**FIGURE 7:**
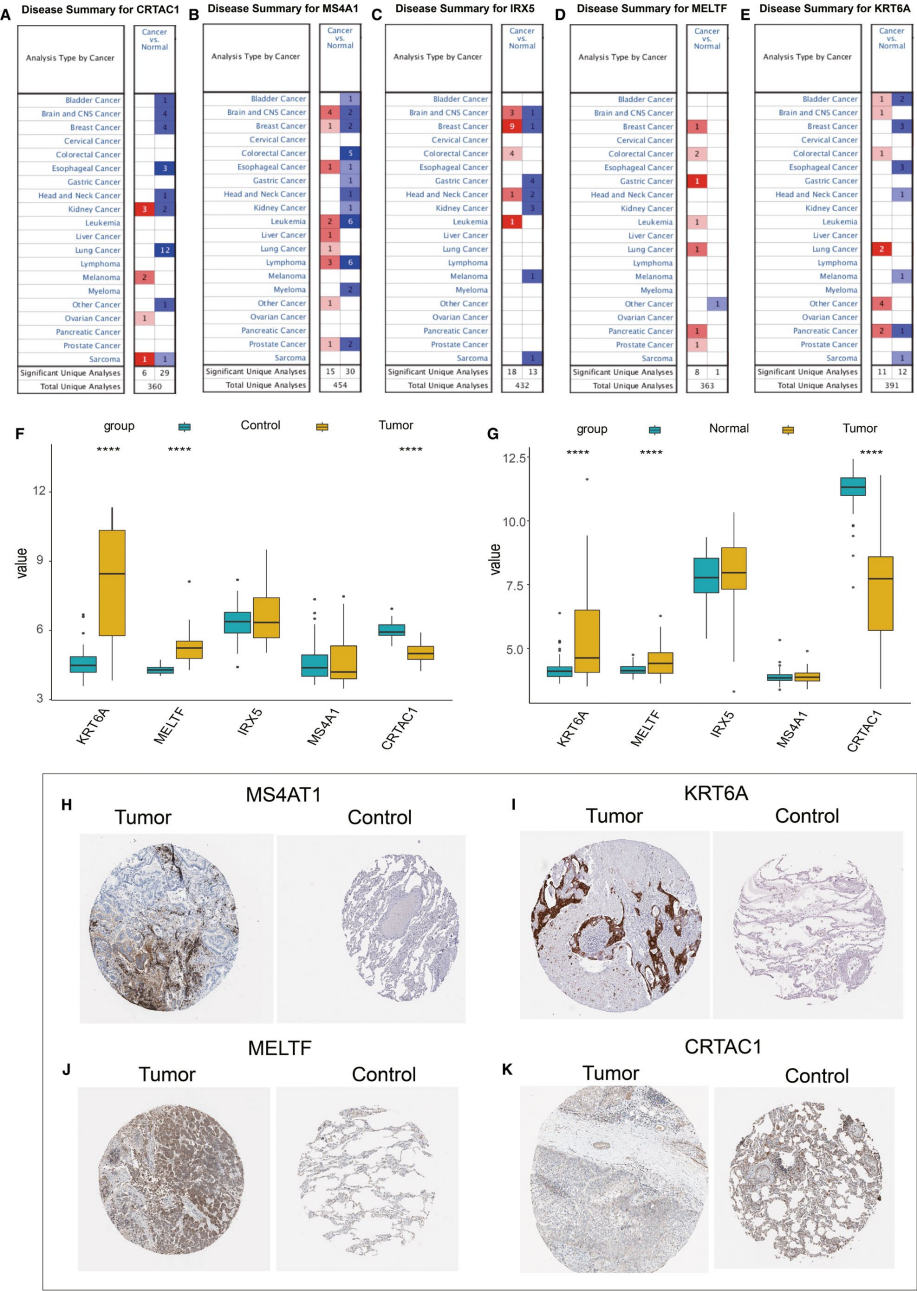
Expression box diagrams of gene expression in pan‐cancer. Expression of *MS4A1* (A), *KRT6A* (B), *MELTF* (C), *CRTAC1* (D) and *IRX5* (E) in different tumours. F, Expression box graph of the 5 genes in the GSE75037 cohort. G, Expression box graph of the 5 genes in the GSE18842 cohort. H, MS4A1 protein expression in cancer and normal control samples. I, KRT6A protein expression in cancer and normal control samples. J, MELTF protein expression in cancer and normal control samples. K, CRTAC1 protein expression in cancer and normal control samples

The results showed that in the Oncomine database, *CRAT1* was lowly expressed in 12 LUAD studies, *MS4A1* and *MELTF* were highly expressed in 1 LUAD study, *KRT6A* was highly expressed in 2 LUAD studies, and *IRX5* showed no significant expression in any LUAD study (Figure 7A‐E). The GSE75037 and GSE18842 cohorts were used to verify the 5 genes’ expression levels in cancer and normal samples with the ggplot2 package in R. The box plots showed that *KRT6A* and *MELTF* had significantly high expression levels in the LUAD samples, and *CRTAC1* was expressed lowly in the LUAD samples in both GEO cohorts (Figure 7F‐G). In general, the results of the GEO and Oncomine databases were almost consistent.

In the HPA database, the immunochemistry results of the 5 genes were analysed, but only 4 genes (*MS4A1*, *KRT6A*, *MELTF* and *CRTAC1*) had protein expression data. The results showed that the expression levels of *MS4A1*, *KRT6A* and *MELTF* in tumour tissues were higher than in normal tissues, while *CRTAC1* expression in tumour tissues was lower than in normal tissues (Figure 7H‐K).

## 4 DISCUSSION

In this study, we first genotyped the 500 LUAD samples of the TCGA data set based on 97 invasion‐related genes, and we divided these samples into 3 subtypes, among which there were significant differences in prognosis. The C3 subtype had poor prognosis, and this was closely related to the pathways of tumorigenesis and development. A total of 669 DEGs were identified, and 5 target genes, including *KRT6A*, *MELTF*, *IRX5*, *MS4A1* and *CRTAC1*, were obtained by using Lasso regression and the AIC algorithm. A 5‐gene prognostic risk model was constructed. The KRT6A protein is a type II cytokeratin, and the *KRT6A* gene is highly expressed in different types of cancer.^25^, ^26^ Some studies have shown that *KRT6A* is overexpressed in LUAD, and the overexpression of *KRT6A* is positively correlated with positive lymph nodes and invasive tumours. High expression of *KRT6A* in LUAD may promote the proliferation and metastasis of lung cancer through epithelial‐mesenchymal transformation and cancer cell transformation.^27^ The KRT6A protein is a potential biomarker for distinguishing LUAD from squamous cell carcinoma.^28^ The IRX5 is a transcription factor that is closely associated with a variety of malignancies.^29^, ^30^ IRX5 can promote the invasion and migration of colorectal cancer cells by inhibiting the RHOA‐ROCK1‐LIMK1 axis.^31^ *IRX5* expression has been shown to be positively correlated with OS in smokers and negatively correlated with OS in non‐smokers with LUAD.^32^ *MS4A1* can be used as an immune‐related gene to predict the prognoses of patients with LUAD,^33^ and the dysregulation of the MS4A1 protein in interstitial lymphocytes may be involved in the progression of asbestos‐related squamous cell carcinoma.^34^ Tissue and serum MELTF levels can be used as biomarkers of gastric cancer progression, and inhibition of *MELTF* expression can inhibit the invasive ability of gastric cancer cells.^35^ Cartilage acidic protein 1 (CRTAC1) is the extracellular matrix protein of human cartilage. CRTAC1 secreted by chondrocytes is the glycosylated extracellular matrix molecule of human articular cartilage.^36^ At present, there have been no studies of MELTF and CRTAC1 in LUAD, but such studies may provide new findings for prognostic markers of LUAD. We plan to further verify the mechanism of MELTF and CRTAC1 in LUAD.

We Z‐scored the risk scores and divided the samples whose risk scores were >0 into the high‐risk group and those whose risk scores were <0 into the low‐risk group. The results showed that the high‐risk score samples had significantly shorter survival times than the low‐risk score samples. By analysing the relationships between risk scores and pathways, we found that the tumour‐related pathways of KEGG_P53_SIGNALING_PATHWAY, KEGG_CELL_CYCLE, KEGG_MISMATCH_REPAIR and KEGG_DNA_REPLICATION increased with increased risk scores. The main ways to repair DNA include base excision repair, mismatch repair, nucleotide excision repair and homologous recombination repair. DNA mismatch repair defects are important biomarkers for predicting the efficacy of immune checkpoint inhibitors in the treatment of many malignant tumours.^37^ Some genes, such as *MCM4*, *MCM5* and *MCM8*, may affect LUAD prognosis by regulating the cell cycle, DNA replication and other biological processes and pathways.^38^ However, the relationships between *KRT6A*, *MELTF*, *IRX5*, *MS4A1* and *CRTAC1* and the p53 signalling pathway, the cell cycle, DNA mismatch repair and DNA replication are still not obvious. Our study may provide new ideas for the study of the mechanism of LUAD progression and metastasis.

According to the significant clinical characteristics in the univariate and multivariate regression analyses, T stage, N stage and risk scores were used to construct the nomogram. Calibration and decision curve analysis curves suggested that the model had good prediction performance. Both the internal and external data sets also confirmed that the 5‐gene signature was robust, and it could perform well in the independent data set (GSE31210). Our model performed better than other models of LUAD. One advantage of our model is that targeted sequencing based on particular genes reduces healthcare costs significantly compared with whole‐genome sequencing. Second, we selected invasion‐related genes as the target genes, which is very important for the early diagnosis and prognosis prediction of LUAD. More importantly, in the routine clinical diagnosis and treatment process, patients’ treatment plans and prognoses are largely dependent on pathological stage, the determination of which currently depends on the anatomic location of LUAD, so the biological heterogeneity of patients with LUAD is not currently being fully reflected. The nomogram we constructed can make up for this deficiency and provide a basis for the individualized treatment of patients with LUAD.

Gene expression was explored by using the Oncomine, GEO and HPA databases. The results showed that the expression levels of *MS4A1* and *KRT6A* in tumour tissues were higher than in normal tissues, while *CRTAC1* expression in tumour tissues was lower than in normal tissues.

Our study has some limitations. First, the population in the TCGA database is predominantly White and Black, and our results need to be validated in other racial groups. Second, the construction of the alignment map was done retrospectively, so our results need to be further validated in multi‐centre clinical trials and prospective studies. In the future, we will explore whether other regression modelling methods can further improve the prediction accuracy of the model.

In conclusion, we identified molecular subtypes of LUAD based on tumour invasion‐related genes, and we developed a 5‐gene signature prognostic hierarchical system. We recommend the use of this classifier as a molecular diagnostic test to assess the prognostic risk of LUAD.

## AUTHOR CONTRIBUTION

**Ping Yu:** Conceptualization (equal); Data curation (equal); Formal analysis (equal); Methodology (equal); Software (equal); Writing‐original draft (equal). **Linlin Tong:** Data curation (equal); Formal analysis (equal); Software (equal); Validation (equal); Writing‐original draft (equal). **Yujia Song:** Investigation (equal); Methodology (equal); Software (equal); Visualization (equal); Writing‐review & editing (equal). **Hui Qu:** Formal analysis (equal); Visualization (equal); Writing‐review & editing (equal). **Ying Chen:** Conceptualization (equal); Funding acquisition (equal); Project administration (lead); Supervision (lead); Validation (lead).

## Supporting information

FigureS1

FigureS2

FigureS3

FigureS4

FigureS5

FigureS6

FigureS7

TableS1

TableS2

TableS3

TableS4
