# Supplementary figures and images for: Systematic profiling of invasion‐related gene signature predicts prognostic features of lung adenocarcinoma
Source: J Cell Mol Med. 2021 May 31;25(13):6388–402. doi: 10.1111/jcmm.16619 (PMC8256358; doi:10.1111/jcmm.16619)

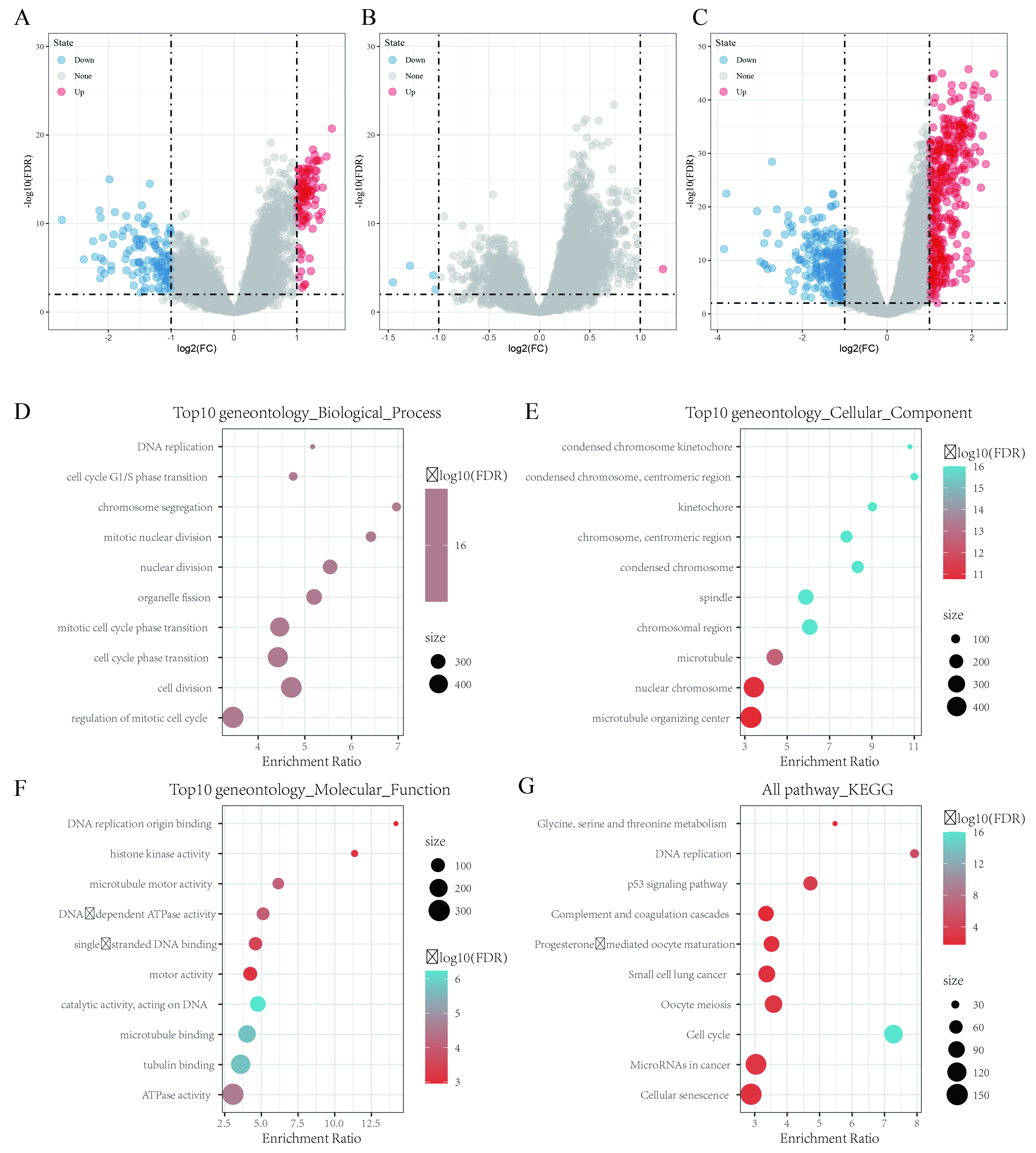

Supplement: Supplementary file 1 — FigureS1 [file JCMM-25-6388-s001.jpg]

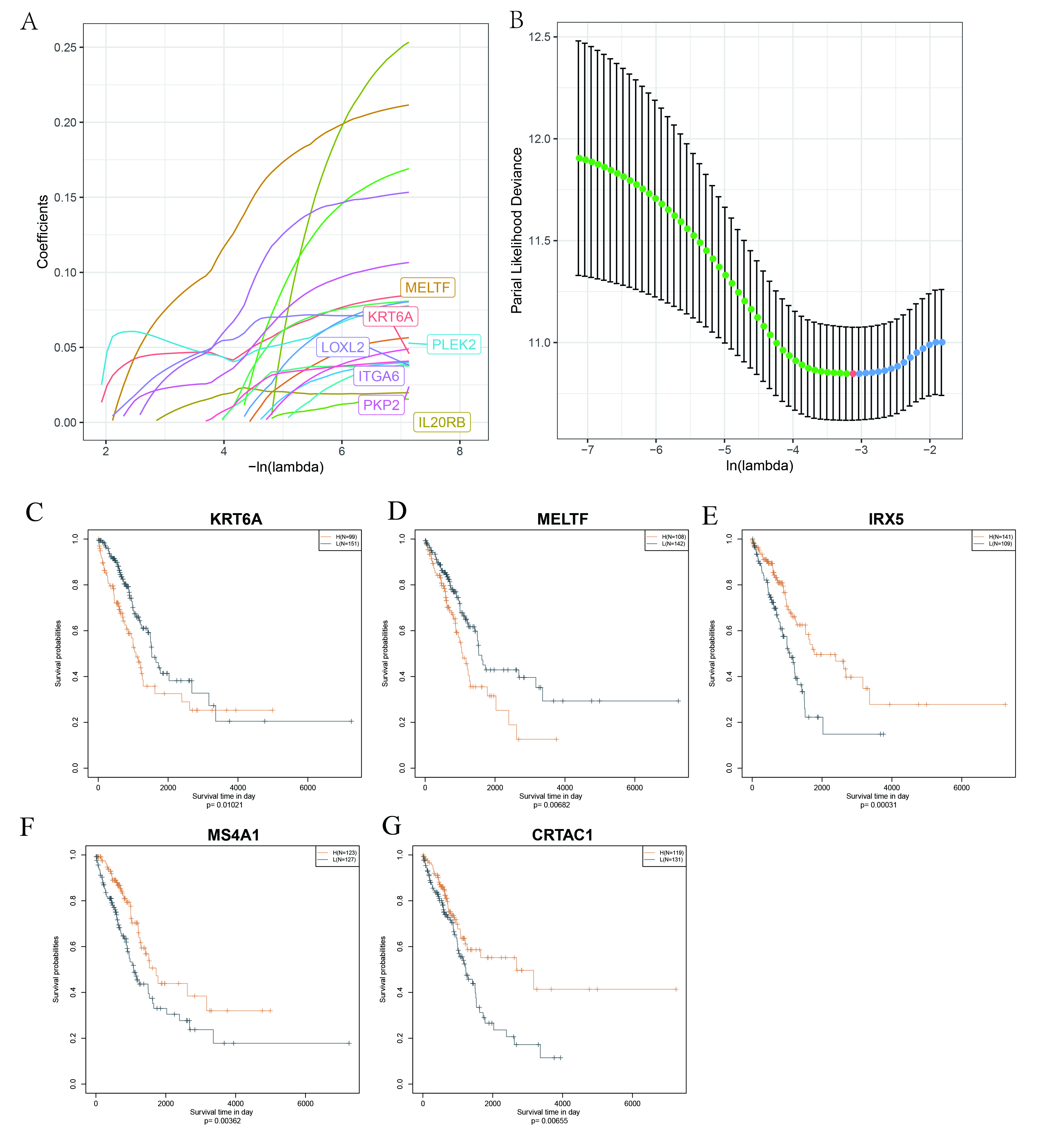

Supplement: Supplementary file 2 — FigureS2 [file JCMM-25-6388-s004.jpg]

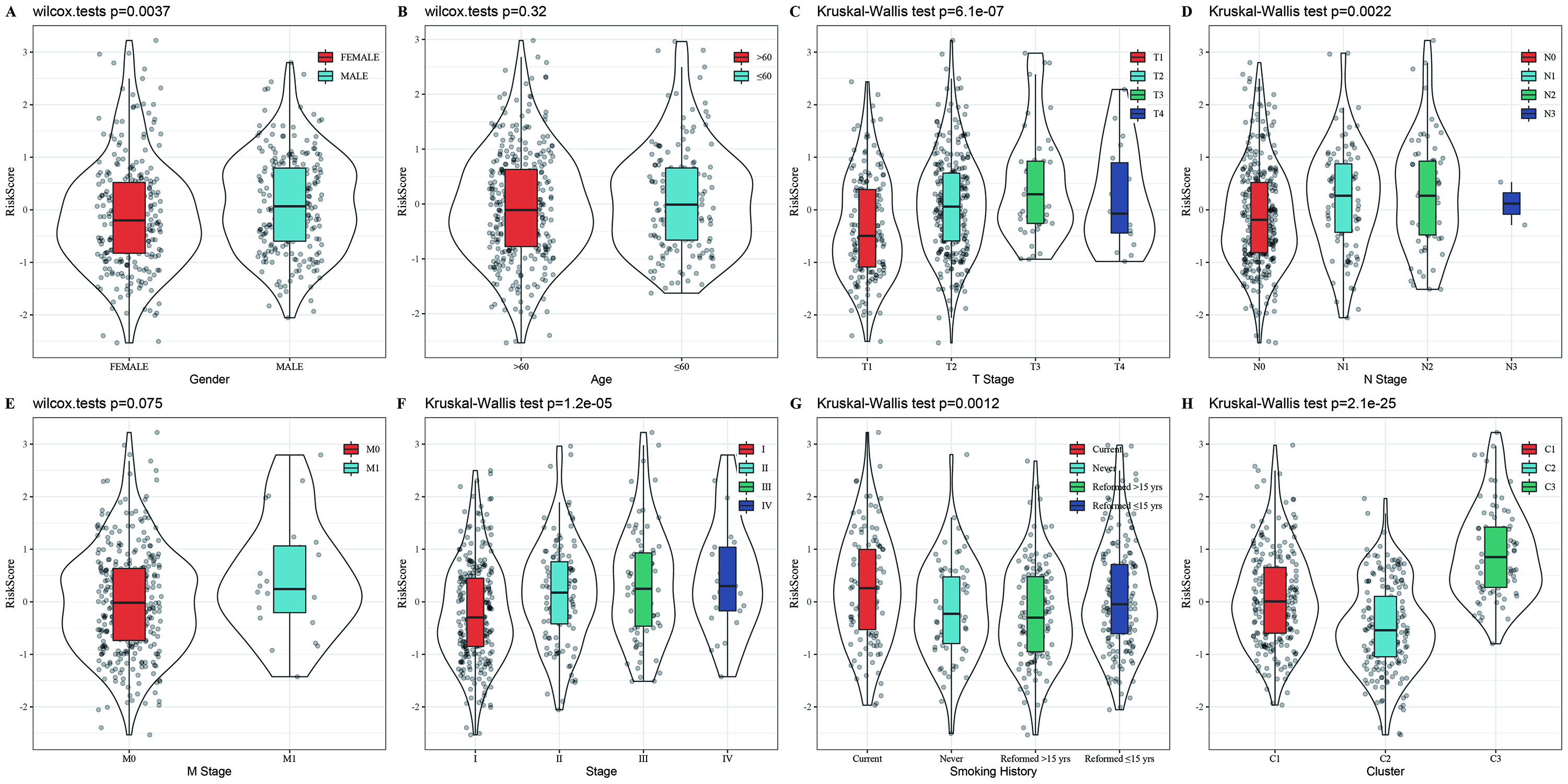

Supplement: Supplementary file 3 — FigureS3 [file JCMM-25-6388-s010.jpg]

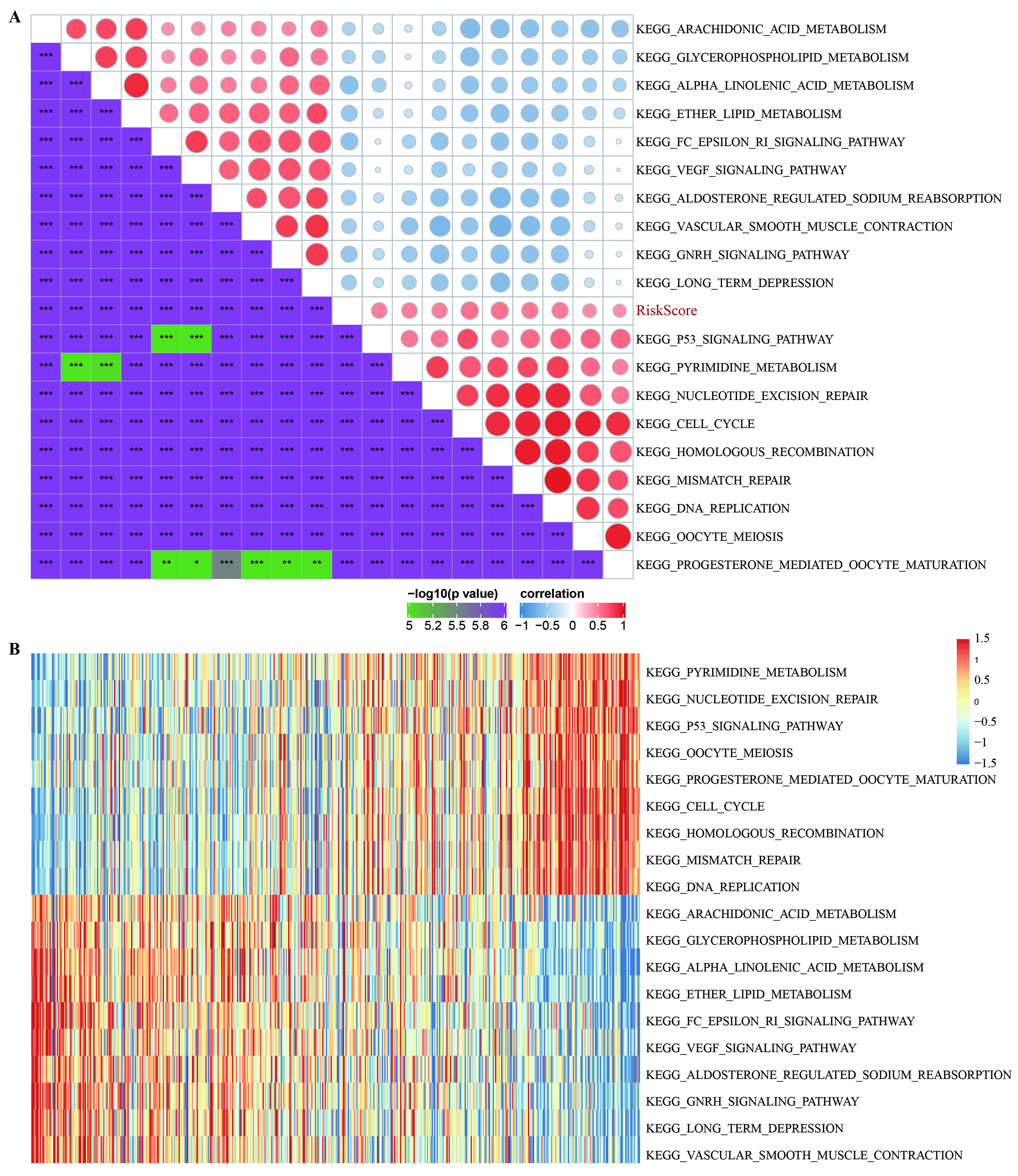

Supplement: Supplementary file 4 — FigureS4 [file JCMM-25-6388-s009.jpg]

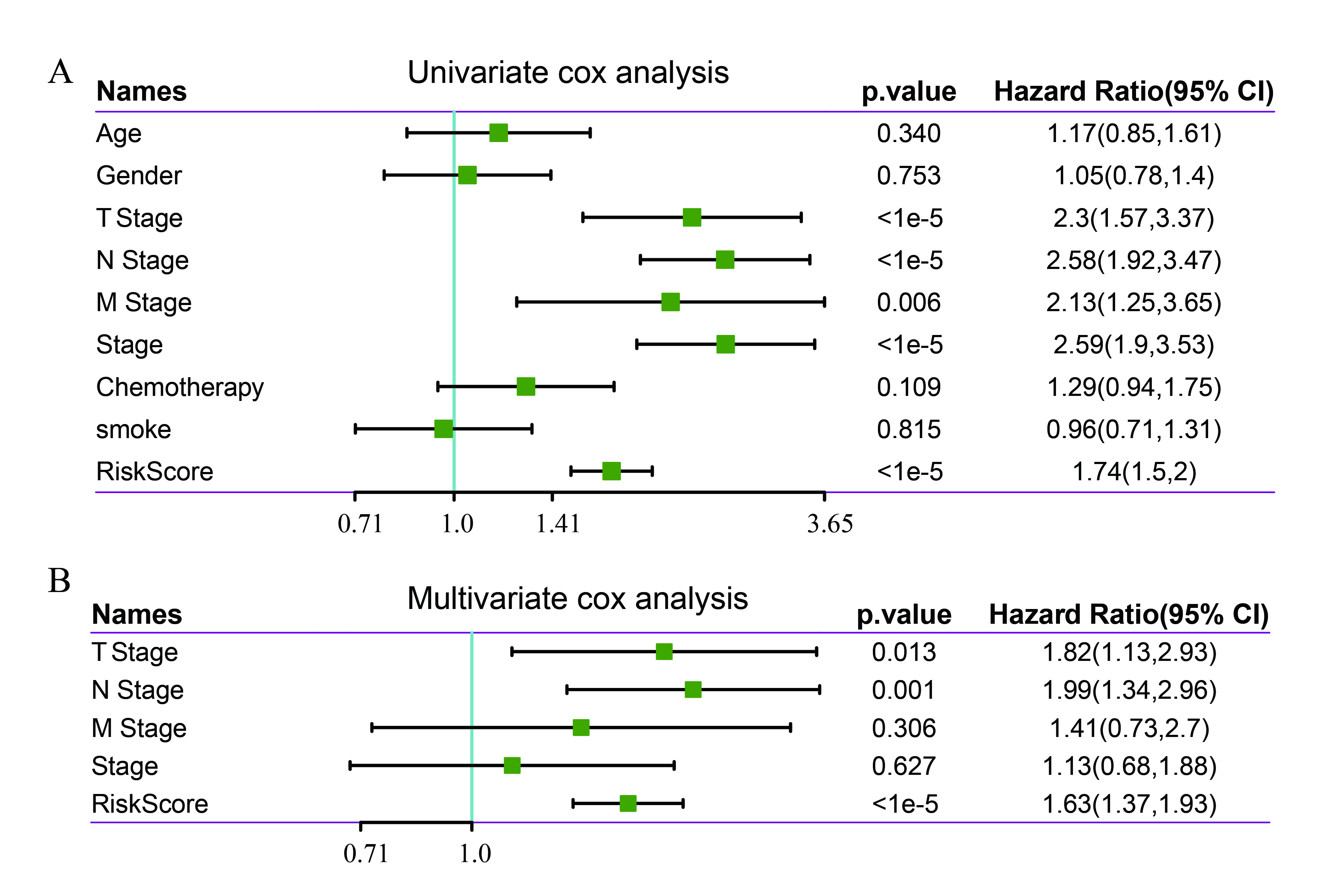

Supplement: Supplementary file 5 — FigureS5 [file JCMM-25-6388-s005.jpg]

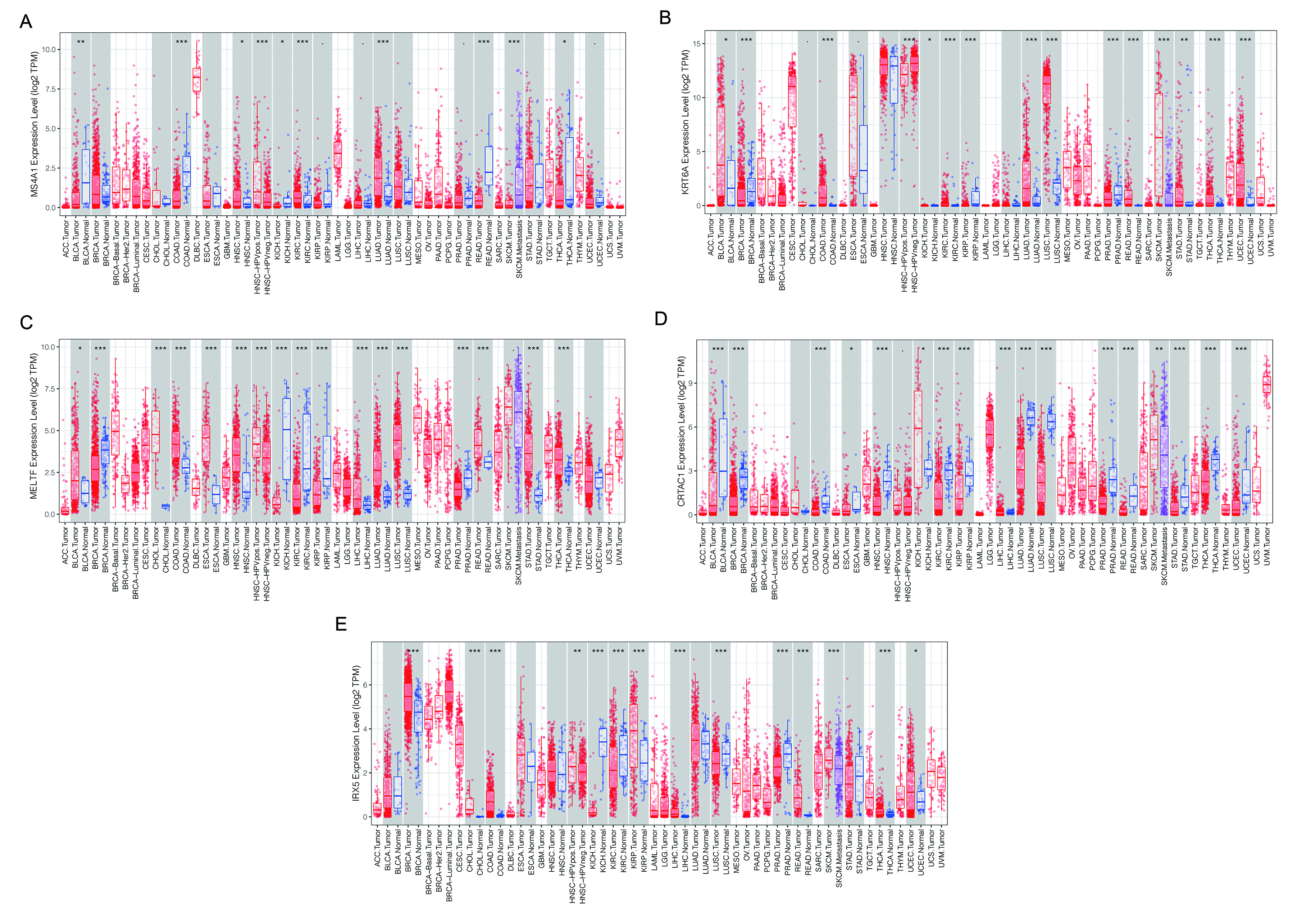

Supplement: Supplementary file 6 — FigureS6 [file JCMM-25-6388-s008.jpg]

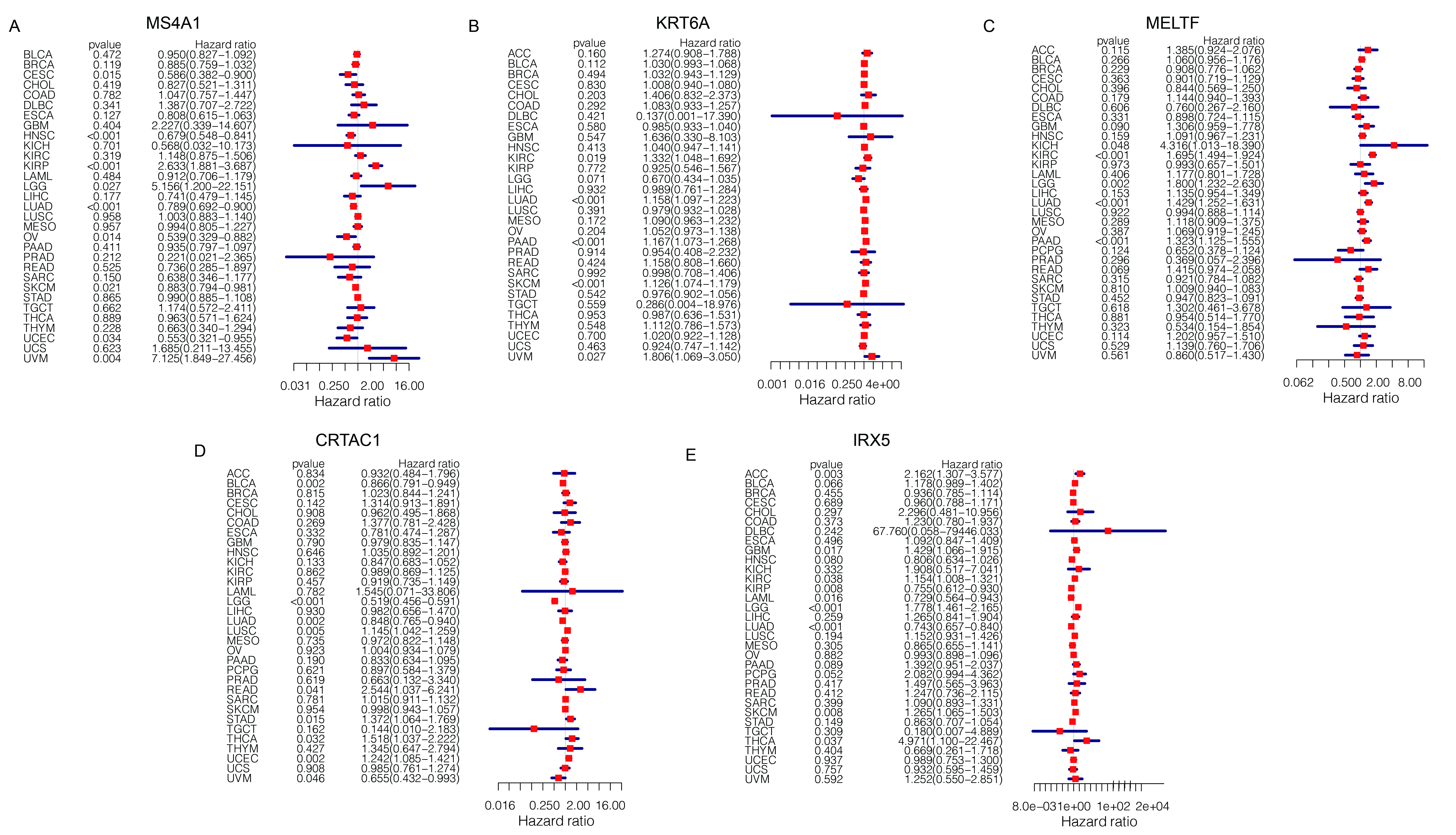

Supplement: Supplementary file 7 — FigureS7 [file JCMM-25-6388-s006.jpg]
