# Supplementary material for: Systematic profiling of invasion‐related gene signature predicts prognostic features of lung adenocarcinoma
Source: J Cell Mol Med. 2021 May 31;25(13):6388–402. doi: 10.1111/jcmm.16619 (PMC8256358; doi:10.1111/jcmm.16619)
Supplement: Supplementary file 8 — TableS1 [file JCMM-25-6388-s011.docx]

Supplementary Table 1. Invasion-related genes were obtained from the CancerSEA website

| **Gene** | **p.value** | **HR** | **Low 95%CI** | **High 95%CI** |
| --- | --- | --- | --- | --- |
| AEBP1 | 0.46523694 | 1.0002029 | 0.99965842 | 1.000747674 |
| AKR1B1 | 0.63922085 | 1.00031602 | 0.998995424 | 1.001638367 |
| AMD1 | 0.99901786 | 0.99999417 | 0.990759111 | 1.009315317 |
| SLC25A5 | 0.30420787 | 1.00020565 | 0.999813391 | 1.00059806 |
| ATP5PB | 0.13152147 | 1.0025657 | 0.999232597 | 1.005909919 |
| BAG1 | 0.56526952 | 1.00154626 | 0.996285705 | 1.006834599 |
| BGN | 0.27518033 | 1.00011827 | 0.999905847 | 1.000330747 |
| C1QB | 0.72532808 | 0.999932 | 0.999552782 | 1.000311371 |
| CALD1 | 0.18256587 | 1.00200685 | 0.999057256 | 1.00496516 |
| CAPG | 0.25688991 | 1.00054648 | 0.999601965 | 1.001491882 |
| CCNE1 | 0.20364088 | 1.00319408 | 0.998273806 | 1.008138612 |
| CDH11 | 0.41569547 | 1.00230327 | 0.996765789 | 1.007871508 |
| CKS1B | 0.0033186 | 1.00524365 | 1.001740827 | 1.008758724 |
| CKS2 | 0.13107088 | 1.00069232 | 0.999793726 | 1.001591716 |
| COL1A1 | 0.0661505 | 1.00009205 | 0.99999386 | 1.00019026 |
| COL1A2 | 0.06493089 | 1.00017297 | 0.999989296 | 1.000356674 |
| COL3A1 | 0.15106697 | 1.00007478 | 0.9999727 | 1.000176868 |
| COL5A1 | 0.04661691 | 1.00123084 | 1.000018423 | 1.002444737 |
| COL5A2 | 0.05850701 | 1.00108396 | 0.999961013 | 1.002208173 |
| COL6A2 | 0.01017447 | 1.00074042 | 1.000175667 | 1.001305483 |
| COL6A3 | 0.18080705 | 1.0007947 | 0.999631042 | 1.001959705 |
| COL10A1 | 0.69596567 | 1.00036866 | 0.998520969 | 1.002219771 |
| COL11A1 | 0.20527193 | 1.00136498 | 0.99925369 | 1.00348074 |
| COMP | 0.84097232 | 0.99981347 | 0.997993238 | 1.001637018 |
| CSE1L | 0.00639161 | 1.00312076 | 1.000876794 | 1.005369751 |
| VCAN | 0.29917537 | 1.00093647 | 0.999169301 | 1.002706755 |
| CTSK | 0.84425399 | 1.00010993 | 0.999013723 | 1.001207344 |
| DAB2 | 0.34722676 | 0.99629635 | 0.988617889 | 1.004034441 |
| DDX5 | 0.26872446 | 0.99906288 | 0.997404347 | 1.000724165 |
| EDNRA | 0.31256699 | 1.0043136 | 0.995958484 | 1.012738806 |
| FAP | 0.38888178 | 1.00466086 | 0.994090272 | 1.015343845 |
| FBN1 | 0.22315167 | 1.00315452 | 0.998084026 | 1.008250779 |
| FN1 | 0.43380959 | 1.00006597 | 0.999900778 | 1.000231192 |
| GNAS | 0.75222806 | 0.99986694 | 0.999041238 | 1.000693317 |
| H2AFZ | 0.0002015 | 1.00177817 | 1.000840186 | 1.002717037 |
| HMGB2 | 0.04510231 | 1.0016048 | 1.000035001 | 1.003177061 |
| HNRNPU | 0.01401298 | 1.0029504 | 1.00059608 | 1.005310258 |
| HSD17B4 | 0.08856965 | 0.9938633 | 0.986847196 | 1.00092928 |
| CCN1 | 0.9888605 | 1.00000479 | 0.999333164 | 1.000676859 |
| INHBA | 0.15291682 | 1.0035377 | 0.998689731 | 1.0084092 |
| LAMB1 | 4.00E-05 | 1.00538663 | 1.002812832 | 1.007967039 |
| LAMC1 | 0.00485906 | 1.0017151 | 1.00052116 | 1.002910459 |
| LGALS1 | 0.26415899 | 1.00007759 | 0.999941405 | 1.000213786 |
| LOX | 0.01943823 | 1.00426416 | 1.000686753 | 1.007854363 |
| LOXL2 | 8.56E-11 | 1.00474672 | 1.00331096 | 1.006184532 |
| LUM | 0.83954878 | 0.99997084 | 0.99968863 | 1.000253133 |
| MMP2 | 0.54005347 | 1.00020502 | 0.999549371 | 1.000861093 |
| MMP11 | 0.83131135 | 1.00008639 | 0.999291776 | 1.000881645 |
| HNRNPM | 0.00340333 | 1.00550845 | 1.001818759 | 1.009211739 |
| NDUFB7 | 0.97124999 | 0.99998412 | 0.999120887 | 1.000848098 |
| YBX1 | 0.00481675 | 1.00067023 | 1.000204197 | 1.001136471 |
| PDGFRB | 0.66450401 | 1.00078462 | 0.997243718 | 1.004338087 |
| PLAU | 0.03236172 | 1.00044562 | 1.000037466 | 1.000853949 |
| PRRX1 | 0.00718963 | 1.0079105 | 1.002136234 | 1.01371804 |
| PNN | 0.76420232 | 1.00042905 | 0.997628981 | 1.003236981 |
| PPIC | 0.00553722 | 1.00532112 | 1.001558564 | 1.00909782 |
| PROS1 | 0.89128018 | 1.00040035 | 0.994674946 | 1.006158702 |
| PSMA2 | 0.00537562 | 1.04269885 | 1.012448868 | 1.073852634 |
| PSMB4 | 0.06093401 | 1.00076515 | 0.999964904 | 1.001566036 |
| RGS4 | 0.99330443 | 0.99994697 | 0.987637679 | 1.012409672 |
| SNAI2 | 1.68E-09 | 1.00849739 | 1.005725866 | 1.011276548 |
| SPOCK1 | 2.01E-06 | 1.01233716 | 1.007231092 | 1.01746911 |
| TGFBI | 0.00307668 | 1.00068214 | 1.000230404 | 1.001134078 |
| THBS2 | 0.10885254 | 1.00065325 | 0.999854773 | 1.001452374 |
| THY1 | 0.14622549 | 1.00230081 | 0.999198458 | 1.005412796 |
| TNFAIP6 | 0.0321319 | 1.01076449 | 1.000913841 | 1.020712084 |
| UBE2V2 | 0.00881767 | 1.01304635 | 1.003267194 | 1.022920825 |
| ADAM12 | 0.01881191 | 1.01287072 | 1.002121343 | 1.023735406 |
| MFAP5 | 0.12039193 | 1.00299991 | 0.999215676 | 1.006798468 |
| ITGBL1 | 0.49207239 | 0.99605242 | 0.984875962 | 1.007355717 |
| TP53I3 | 0.00200047 | 1.00451143 | 1.001647659 | 1.007383387 |
| NUAK1 | 0.75584005 | 1.00212739 | 0.988793308 | 1.015641284 |
| HNRNPDL | 0.70274539 | 1.00079584 | 0.996715152 | 1.004893242 |
| TXNDC9 | 0.08849231 | 1.00623381 | 0.999064444 | 1.013454628 |
| LRRC17 | 0.29447662 | 1.00901268 | 0.99222833 | 1.026080952 |
| IFI30 | 0.30346807 | 0.96569128 | 0.903566501 | 1.032087449 |
| POSTN | 0.02586836 | 1.00086869 | 1.00010453 | 1.001633443 |
| CBX1 | 0.04356665 | 1.00311226 | 1.000089724 | 1.00614393 |
| NID2 | 0.00149043 | 1.01828674 | 1.006964474 | 1.029736308 |
| RRAS2 | 0.79875652 | 0.99939482 | 0.994754707 | 1.004056568 |
| RALY | 0.99639669 | 0.99998969 | 0.995523714 | 1.004475694 |
| SEPHS2 | 0.38263657 | 1.00070206 | 0.999126646 | 1.00227996 |
| HEY1 | 0.97336502 | 1.00011189 | 0.993564893 | 1.006702026 |
| MXRA5 | 0.04324862 | 1.00201083 | 1.00006096 | 1.003964504 |
| OLFML2B | 0.00081294 | 1.00588673 | 1.00243673 | 1.009348606 |
| TMEM158 | 0.02891442 | 1.01344325 | 1.001374346 | 1.025657605 |
| WWTR1 | 0.22262947 | 1.0042958 | 0.997400987 | 1.011238285 |
| GREM1 | 0.14709476 | 1.00376026 | 0.998680415 | 1.00886595 |
| NOX4 | 0.36119877 | 1.02444613 | 0.972688682 | 1.078957628 |
| CLEC4A | 0.0478267 | 0.98573474 | 0.971806614 | 0.999862487 |
| COPZ2 | 0.43601107 | 1.00405983 | 0.993875888 | 1.014348127 |
| ASPN | 0.6090114 | 1.00073251 | 0.997928512 | 1.003544383 |
| CEMIP | 0.2339937 | 1.00108905 | 0.999296186 | 1.002885133 |
| CRISPLD2 | 0.84724018 | 1.00080047 | 0.992686164 | 1.008981101 |
| TUBB6 | 1.81E-06 | 1.00949955 | 1.005588557 | 1.01342576 |
| LRRC15 | 0.57393724 | 1.00166949 | 0.995861949 | 1.007510891 |
| TUBB | 0.02606193 | 1.00036356 | 1.00004334 | 1.000683877 |
