# Supplementary material for: Systematic profiling of invasion‐related gene signature predicts prognostic features of lung adenocarcinoma
Source: J Cell Mol Med. 2021 May 31;25(13):6388–402. doi: 10.1111/jcmm.16619 (PMC8256358; doi:10.1111/jcmm.16619)
Supplement: Supplementary file 9 — TableS2 [file JCMM-25-6388-s002.docx]

Supplementary Table 2. 19 genes were found to be associated with LUAD prognosis

| **Gene** | **p.value** | **HR** | **Low 95%CI** | **High 95%CI** |
| --- | --- | --- | --- | --- |
| CKS1B | 0.0033186 | 1.00524365 | 1.001740827 | 1.008758724 |
| CSE1L | 0.00639161 | 1.00312076 | 1.000876794 | 1.005369751 |
| H2AFZ | 0.0002015 | 1.00177817 | 1.000840186 | 1.002717037 |
| LAMB1 | 4.00E-05 | 1.00538663 | 1.002812832 | 1.007967039 |
| LAMC1 | 0.00485906 | 1.0017151 | 1.00052116 | 1.002910459 |
| LOXL2 | 8.56E-11 | 1.00474672 | 1.00331096 | 1.006184532 |
| HNRNPM | 0.00340333 | 1.00550845 | 1.001818759 | 1.009211739 |
| YBX1 | 0.00481675 | 1.00067023 | 1.000204197 | 1.001136471 |
| PRRX1 | 0.00718963 | 1.0079105 | 1.002136234 | 1.01371804 |
| PPIC | 0.00553722 | 1.00532112 | 1.001558564 | 1.00909782 |
| PSMA2 | 0.00537562 | 1.04269885 | 1.012448868 | 1.073852634 |
| SNAI2 | 1.68E-09 | 1.00849739 | 1.005725866 | 1.011276548 |
| SPOCK1 | 2.01E-06 | 1.01233716 | 1.007231092 | 1.01746911 |
| TGFBI | 0.00307668 | 1.00068214 | 1.000230404 | 1.001134078 |
| UBE2V2 | 0.00881767 | 1.01304635 | 1.003267194 | 1.022920825 |
| TP53I3 | 0.00200047 | 1.00451143 | 1.001647659 | 1.007383387 |
| NID2 | 0.00149043 | 1.01828674 | 1.006964474 | 1.029736308 |
| OLFML2B | 0.00081294 | 1.00588673 | 1.00243673 | 1.009348606 |
| TUBB6 | 1.81E-06 | 1.00949955 | 1.005588557 | 1.01342576 |
