# Supplementary material for: Systematic profiling of invasion‐related gene signature predicts prognostic features of lung adenocarcinoma
Source: J Cell Mol Med. 2021 May 31;25(13):6388–402. doi: 10.1111/jcmm.16619 (PMC8256358; doi:10.1111/jcmm.16619)
Supplement: Supplementary file 11 — TableS4 [file JCMM-25-6388-s007.docx]

Supplementary Table 4. 29 genes associated with prognosis to conduct lasso cox analysis

| **Gene** | **p.value** | **HR** | **Low 95%CI** | **High 95%CI** |
| --- | --- | --- | --- | --- |
| KRT6A | 5.11E-06 | 1.16548294 | 1.091243468 | 1.244773061 |
| ANLN | 0.00079913 | 1.27143625 | 1.104922766 | 1.463043569 |
| SLC16A1 | 0.00035512 | 1.25912333 | 1.109560384 | 1.428846591 |
| NEIL3 | 0.0006108 | 1.30443126 | 1.120483118 | 1.518577913 |
| MELTF | 1.19E-05 | 1.39078629 | 1.199869539 | 1.612080676 |
| IL20RB | 0.00050862 | 1.19074575 | 1.07912106 | 1.313916947 |
| SHCBP1 | 0.00031296 | 1.43812042 | 1.180291385 | 1.752270988 |
| GAPDH | 0.00032745 | 1.54875977 | 1.219932718 | 1.966220586 |
| ECT2 | 0.00044526 | 1.38873694 | 1.156166165 | 1.668090927 |
| VIPR1 | 0.00080496 | 0.72572724 | 0.601649231 | 0.875393839 |
| RGS20 | 0.00024834 | 1.36255047 | 1.154738055 | 1.607761849 |
| PLOD2 | 0.0003617 | 1.28865586 | 1.121017604 | 1.481362942 |
| ARNTL2 | 6.91E-05 | 1.33810067 | 1.159282559 | 1.544501291 |
| PLEK2 | 1.61E-05 | 1.51359081 | 1.25374366 | 1.827293092 |
| FSCN1 | 0.00048927 | 1.30046971 | 1.121912498 | 1.507445068 |
| DSG2 | 0.00063674 | 1.43823154 | 1.167504698 | 1.771735875 |
| GNA14 | 0.00084102 | 0.75170961 | 0.635756802 | 0.888810531 |
| LOXL2 | 4.44E-05 | 1.37585676 | 1.180474418 | 1.603577169 |
| TMEM171 | 0.00053569 | 1.37936108 | 1.14975877 | 1.654814076 |
| IRX5 | 5.30E-05 | 0.74394409 | 0.644541382 | 0.858676914 |
| SLC16A11 | 0.0004521 | 0.68734192 | 0.557429398 | 0.847531384 |
| FAM189A2 | 2.95E-05 | 0.69131755 | 0.581371284 | 0.822056349 |
| ITGA6 | 0.00078591 | 1.3222419 | 1.123309046 | 1.556404852 |
| PKP2 | 0.00021853 | 1.29231173 | 1.128026039 | 1.480523985 |
| MS4A1 | 0.00065048 | 0.78617136 | 0.684632392 | 0.902769731 |
| BMP5 | 0.00056089 | 0.78216442 | 0.680264098 | 0.899328924 |
| GJB3 | 0.00043744 | 1.20691166 | 1.086804316 | 1.340292575 |
| GJB2 | 0.00053163 | 1.23192691 | 1.094801626 | 1.386227316 |
| CRTAC1 | 0.00027841 | 0.81318197 | 0.727371536 | 0.909115751 |
